# Impaired coronary vasodilation with Regadenoson in patients with angiographically normal coronaries when compared to normal volunteers - Insights from Quantitative MRI Perfusion

**DOI:** 10.1186/1532-429X-13-S1-O66

**Published:** 2011-02-02

**Authors:** Sujethra Vasu, W Patricia Bandettini, Li-Yueh Hsu, Peter Kellman, Marcus Y Chen, Joel Wilson, Steve Leung, Sujata M Shanbhag, O Julian Booker, Christine Mancini, Jennifer Henry, Tracy Lowrey, Andrew E Arai

**Affiliations:** 1 National Institutes of Health, Bethesda, MD, USA

## Introduction

Quantitative myocardial perfusion to assess impaired coronary vasoreactivity has been used to identify subclinical atherosclerosis. Impaired coronary vasodilation has been observed in patients with cardiac risk factors in the MESA cohort. The coronary vasodilator response in patients with angiographically normal coronary arteries/minimal stenosis is not known. The purpose of this study was to compare the stress myocardial blood flow (MBF) of patients with normal coronaries/minimal stenosis with normal volunteers.

## Hypothesis

Patients with angiographically normal coronaries/minimal stenosis have impaired vasodilator response when compared to normal volunteers.

## Methods

Twenty patients with normal coronaries/minimal stenosis on coronary CT angiography had also undergone stress MR with regadenoson. Seventeen healthy normal volunteers with Framingham score less than 1% underwent stress testing with regadenoson. Using a SSFP perfusion sequence, stress imaging was done 70 seconds post regadenoson injection. All patients and volunteers received aminophylline after stress imaging. Rest imaging was done 20 minutes later. Myocardial blood flow (MBF) in ml/min/g and myocardial perfusion reserve (MPR) were quantified using a fully quantitative model constrained deconvolution (MCD).

## Results

**Figure 1:**
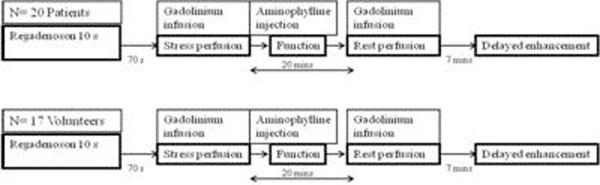
Study Design

**Figure 2:**
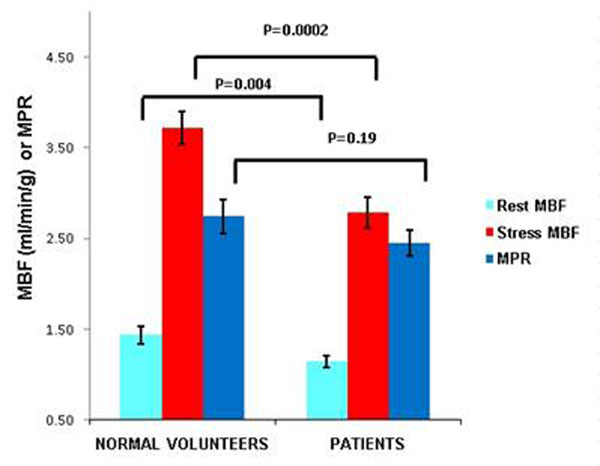
Rest, Stress MBF and MPR between normal volunteers and patients,

Stress MBF (mean ± standard error) was higher in normal volunteers (3.72 ± 0.18) than patients (2.78 ± 0.14), p=.0002. When stratified by risk factors (0-1, >1) the stress MBF of patients with >1 risk factor was significantly lower than young, healthy, normal volunteers (2.64± 0.12 vs. 3.72± 0.18, p= 0.00005). Figures 1 and 2.

**Figure 3:**
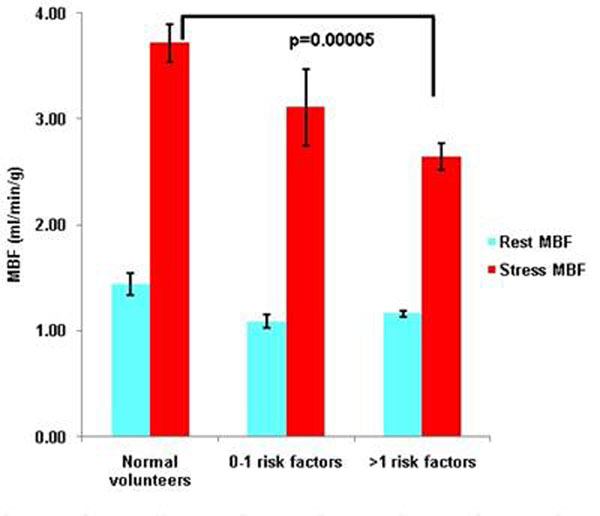
Rest and Stress MBF in normal volunteers and patients stratified by risk factors.

## Conclusions

Despite angiographically normal coronaries/minimal stenosis on CT, patients referred for stress testing have impaired coronary vasodilator response relative to young, healthy normal volunteers. The magnitude of this effect is larger in patients with >1 risk factor.

